# Case of *Fonsecaea nubica* chromoblastomycosis from the French territory of Mayotte

**DOI:** 10.1099/jmmcr.0.004218

**Published:** 2014-12-01

**Authors:** Estelle Cateau, Vincent Cante, Dea Garcia Hermoso, Marie-Helene Rodier

**Affiliations:** ^1^​ Laboratoire de Parasitologie et Mycologie Medicale, CHU de Poitiers, 2 rue de la Miletrie, BP 577 86021 Poitiers, France; ^2^​ Service de Dermatologie, CHU de Poitiers, 2 rue de la Miletrie, BP 577 86021 Poitiers, France; ^3^​ Unité de Mycologie Moléculaire, Centre National Mycoses Invasives et Antifongiques, Institut Pasteur, Paris, France; ^4^​ CNRS URA3012, Paris, France

**Keywords:** chromoblastomycosis, cutaneous lesions, itraconazole

## Abstract

**Introduction:**

*Fonsecaea nubica* is a newly described species in the genus *Fonsecaea*.

**Case presentation:**

We describe here a clinical case of chromoblastomycosis in a 66-year-old man who presented a 3-year history of leg lesions. *F. nubica* was identified by morphological and molecular methods. Four months of treatment with itraconazole (300 mg daily) significantly improved the lesions.

**Conclusion:**

To the best of our knowledge, this case represents the first clinical case of *F. nubica* described in France. Particular attention should be paid to microscopic examination for fungal cultures in order to avoid confusion with a contaminating fungus. Moreover, recurrent, wide and deep biopsies should be performed to monitor the evolution of the lesions.

## Introduction

*Fonsecaea nubica* is a new species in the genus *Fonsecaea*, first described in 2010. This species is morphologically similar to *Fonsecaea pedrosoi* and *Fonsecaea monophora*, and is only distinguishable using rDNA gene internal transcribed spacer (ITS) sequence data (Najafzadeh *et al.*, 2010).

To the best of our knowledge, we are reporting the first case of chromoblastomycosis caused by *F. nubica* in France, diagnosed in a resident of Mayotte, a French overseas territory. This species has also been reported in cases of chromoblastomycosis occurring in Laos (Slesak *et al.*, 2011) and in China and South Africa (Najafzadeh *et al.*, 2011; Sun *et al.*, 2012).

## Case report

A 66-year-old man from Mayotte, an overseas department of France located between Africa and Madagascar, presented with lower right leg lesions that had been developing for 3 years. At first, the lesions were ulcerative, and antibiotic treatment had not led to any improvement.

**Fig. 1.**
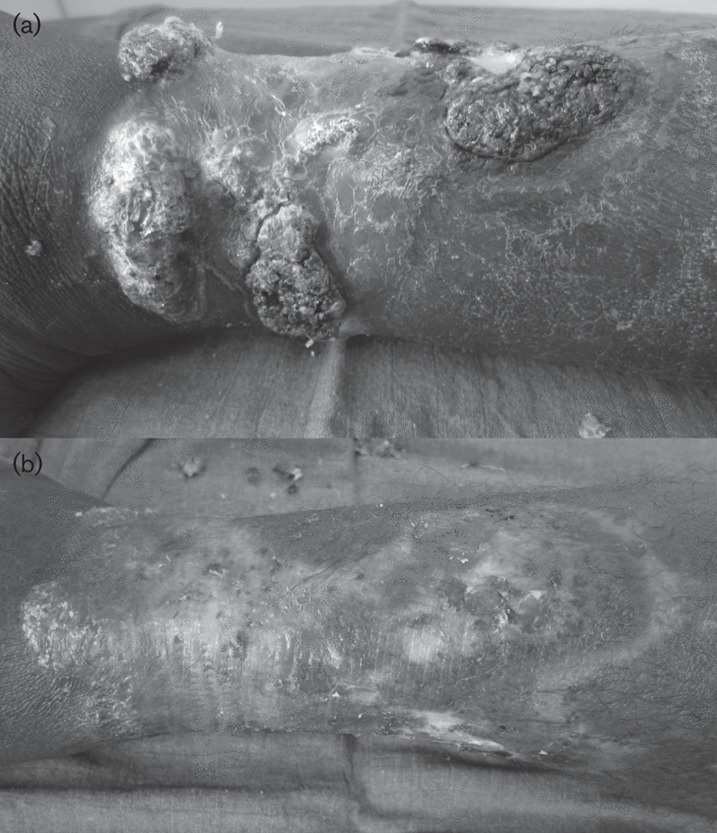
Cauliflower-like lesions on the right lower leg of the patient before (a) and after (b) treatment.

On examination, the lesions were found to be cauliflower-like, purulent and ulcerative (Fig. 1a). A biopsy was performed for bacterial, mycobacterial and fungal cultures. Histological examination of the resected specimen showed no evidence of malignancy, no fungal structure and no sclerotic cells. Given the uncertain diagnosis, a test treatment was carried out with amoxicillin-clavulanic acid *per os* and tulle gras, together with clobetasol daily dressings.

**Fig. 2.**
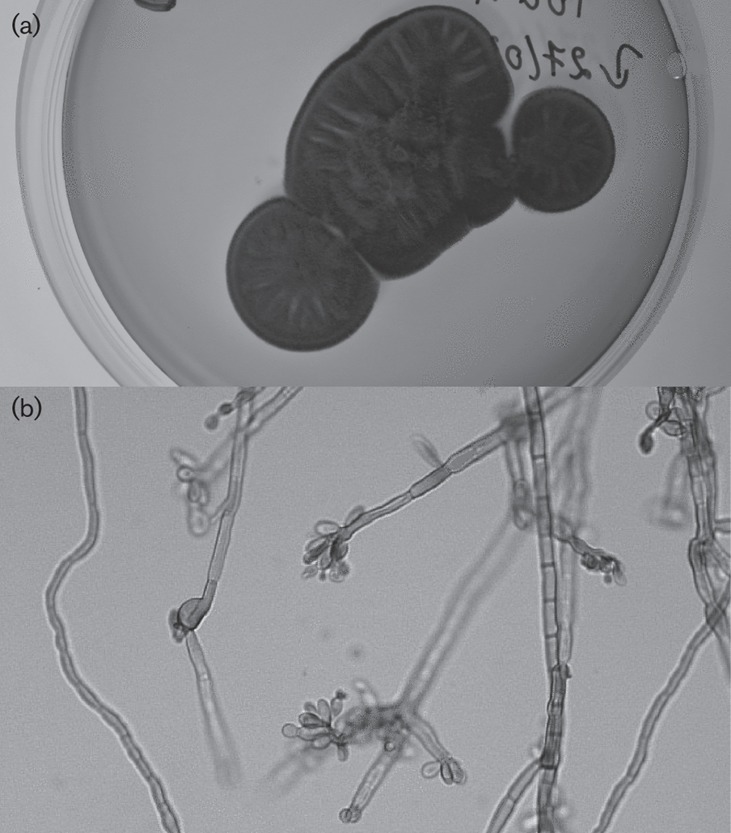
Culture of *F. nubica* on malt extract medium (a) and microscopic examination of the colony in lactophenol blue solution (b).

After 21 days of culture, dark velvety colonies were isolated on Sabouraud chloramphenicol agar slants incubated at 37 °C (Fig. 2a), and a few days later the same colonies appeared on Sabouraud chloramphenicol medium incubated at 27 °C. The filamentous fungus was phenotypically identified as belonging to a member of the genus *Fonsecaea* (Fig. 2b), having septate dark brown hyphae and suberect conidiophores that were highly branched at the apices. The conidiophores were pale brown, septate and sympodial with conidiogenous zones confined to the upper portion. The conidia (1.5–3×2.5–6 µm) arose upon swollen denticles located at the tips of the conidiophores, and they were brown and barrel-shaped. Species-specific molecular identification was ascertained by amplification and sequencing of the internal transcribed spacer 1 (ITS1)–5.8S–ITS2 region of the rDNA gene (Alvarez *et al.*, 2010). A blast (http://www.ncbi.nlm.nih.gov/BLAST) search revealed 99 % identity (565/568 bp) with the type strain of *F. nubica* CBC 269.64.

Antifungal susceptibility was determined by the broth microdilution EUCAST method [Subcommittee on Antifungal Susceptibility Testing (AFST) of the ESCMID European Committee for Antimicrobial Susceptibility Testing (EUCAST), 2008], which was applied with some modifications. MICs for echinocandins (caspofungin, micafungin and anidulafungin) were high (0.25 mg l^−1^), but they were considerably lower for amphotericin B (0.125 mg l^−1^) and for azoles (itraconazole and posaconazole, 0.014 mg l^−1^; voriconazole, 0.003 mg l^−1^).

After 1 month, the patient still presented verrucous lesions near the malleolus. Another biopsy was performed, and cultures resulted in isolation of the same fungus.

Two months after the initial admission, itraconazole treatment was started (300 mg daily) associated with hepatic and haematological monitoring, and 4 months later the lesions were nearly healed.

Ten months after the first diagnosis, two small lesions still persisted (Fig. 1b), and a biopsy was performed. As *F. nubica* was once again recovered in the resected tissue, itraconazole treatment was continued. The patient was seen 16 months after the first diagnosis and still presented budding lesions, so itraconazole treatment (300 mg daily) was continued. The patient was due to be seen again in 3 months’ time.

## Discussion

Chromoblastomycosis is a worldwide chronic infection of the skin and subcutaneous tissue, most commonly found in tropical and subtropical climates. It is caused mainly by dematiaceous fungi such as *Fonsecaea*, *Phialophora* and *Cladophialophora* spp., which are saprophytes in soil and plants (Ameen, 2009). *F. pedrosoi* is the most common agent in tropical rain forests, as is *Cladophialophora carrionii* in dry countries and desert regions. Infections usually result from trauma with contaminated thorns or wood splinters, and the fungi are frequently found in predominantly male farmers, laborers and persons walking barefoot. They most commonly affect the limbs, particularly the lower legs and feet.

Chromoblastomycosis is a slowly developing fungal infection, presenting first as a dermatophyte infection or a papula, and then being transformed into nodules and verrucous lesions. After many years, the lesions may have developed into tumoral, cauliflower-like masses (Queiroz–Telles *et al.*, 2009). Satellite lesions can also appear and may be aggravated by autoinoculation through scratching and from lymphatic dissemination. Bacterial secondary infection is a common complication (Ameen, 2009).

Diagnosis is based on direct microscopy and culture. Microscopic examination of scrapings taken from the lesion reveals multiseptate sclerotic cells (muriform cells, commonly known as “copper pennies”) that are pathognomonic of chromoblastomycosis. These elements can also be recovered on histopathological examination. Cultures are performed on Sabouraud dextrose agar medium, but the filamentous fungi are quite slow growing (Ameen, 2009), which is the reason why culture medium should be incubated for at least a few weeks when chromoblastomycosis is suspected. The fungal colonies present characteristics ranging from flat to heaped and folded, suede-like to downy and olivaceous to black with black reverse. Morphological distinction of *Fonsecaea* spp. is difficult, but their separation on the basis of mutlilocus data is unambiguous (Najafzadeh *et al.*, 2010).

Chromoblastomycosis is associated with low cure rates and high relapse rates, particularly in chronic and extensive disease (Ameen, 2009). Despite being the most common aetiological agent, *F. pedrosoi* appears to be less sensitive to antifungal therapy than *C. carrionii* or *Phialophora verrucosa* (López Martínez & Méndez Tovar, 2007). In patients who present early with small lesions, the goal of treatment should be cure, whereas in cases of extensive lesions, even years of drug treatment may fail to clear them. Generally speaking, clinical cure consists of complete resolution of lesions, whilst mycological cure is defined as negative direct examination and culture. Long-term disease management involves extended courses of antifungal chemotherapy, which are often combined with physical treatments such as surgery, cryotherapy or thermotherapy. To date, itraconazole (200–400 mg daily) and terbinafine (500–1000 mg daily) have shown maximal efficacy when treatment is followed up for 6–12 months (Bonifaz *et al.*, 2001; Queiroz–Telles *et al.*, 2003). Pulse itraconazole (400 mg daily for 1 week every month) has been shown to be effective and to increase compliance (Ungpakorn & Reangchainam, 2006). Combination therapy using itraconazole and terbinafine has also shown efficacy because the drugs appear to act synergistically (Gupta *et al.*, 2002). New azoles such as posaconazole and voriconazole seem to be effective in the management of chromoblastomycosis, but reported experiences have been limited to date.

This clinical case highlights the fact that the identification of strains by microbiologists depends not only on their morphology but also on clinical information (e.g. lesion morphology, geographical location of the patient). When chromoblastomycosis is suspected, the culture medium must be incubated for at least 4 weeks between 27 and 37 °C, and in order to avoid confusion with a contaminating fungus, attentive direct examination is required.

Finally, it is necessary to keep in mind that only negativity of mycological cultures can confirm total cure, and that antifungal treatment consequently has to be continued for as long as the fungus can be isolated in culture. More specifically, recurrent, wide and deep biopsies should be performed in the framework of histopathological examination and for fungal cultures.

## Abbreviations

ITS: internal transcribed spacer
